# Profiling the socioeconomic characteristics, dietary intake, and health status of Korean older adults for nutrition plan customization: a comparison of principal component, factor, and cluster analyses

**DOI:** 10.4178/epih.e2024043

**Published:** 2024-04-12

**Authors:** Kyungsook Woo, Kirang Kim

**Affiliations:** 1 Department of Food Science and Nutrition, Dankook University, Cheonan, Korea; 2 Institute of Health and Society, Hanyang University, Seoul, Korea

**Keywords:** Principal component analysis, Factor analysis, Cluster analysis, Aged, Nutritional status

## Abstract

**OBJECTIVES:**

This study was conducted to establish profiles of socioeconomic characteristics, dietary intake, and health status among Korean older adults by employing 3 multivariate analysis techniques.

**METHODS:**

Data were obtained from 1,352 adults aged 65 years and older who participated in the 2019 Korea National Health and Nutrition Examination Survey. Principal component analysis (PCA), factor analysis (FA), and cluster analysis (CA) were utilized for profiling, with data preprocessing undertaken to facilitate these approaches.

**RESULTS:**

PCA, FA, and CA yielded similar results, reflecting the high common variance among the variables. PCA identified 4 components, accounting for 71.6% of the accumulated variance. FA revealed 5 factors, displaying a Kaiser-Meyer-Olkin value of 0.51 and explaining 74.3% of the total variance. Finally, CA grouped the participants into 4 clusters (R^2^=0.465). Both PCA and FA identified dietary intake (energy, protein, carbohydrate, etc.), social support from family (incorporating family structure, number of family numbers, and engagement in social eating), and health status (encompassing oral, physical, and subjective health) as key factors. CA classified Korean older adults into 4 distinct typologies, with significant differences observed in dietary intake, health status, and household income (p<0.01).

**CONCLUSIONS:**

The study utilized PCA, FA, and CA to analyze profiling domains and derive characteristics of older adults in Korea, followed by a comparison of the results. The variables defining the clusters in CA were consistent with those identified by PCA and FA.

## GRAPHICAL ABSTRACT

[Fig f3-epih-46-e2024043]

## Key Message

Profiling analysis using PCA, FA, and CA was performed to characterize and classify older adults in Korea on the same dataset. As a result of the analysis, the three techniques showed similar patterns in the characteristics of Korean older adults.

## INTRODUCTION

Aging is a multidimensional process characterized by a range of physical, social, and physiological changes that humans experience over the course of their lives. A population is described as aging when the proportion aged 65 years and older reaches 7% [1]. In Korea, the demographic landscape is marked by low birth rates and an aging population [2].

Maintaining an adequate nutritional status is a prerequisite for well-being and a good quality of life [3]. The promotion of healthy aging requires a comprehensive approach that includes understanding the demographic and socioeconomic factors, health status, health behaviors, and dietary intake of older adults [4]. Simultaneously, it is important to categorize members of the older population based on relevant characteristics, which may aid in developing policies and strategies to establish personalized nutrition plans supporting their overall well-being [5].

Previous studies have examined nutrient intakes, dietary habits, and food consumption to better understand the dietary patterns of older adults in Korea [6,7]. Additionally, multiple studies have sought to identify characteristics of this demographic beyond their dietary patterns. However, these investigations have generally focused on specific individual aspects, such as lifestyle and health behaviors [8,9], health status [10,11], or social support [12,13]. Few studies have endeavored to integrate a range of factors from diverse domains to characterize this population [14,15].

In epidemiological studies, exploratory statistical approaches are widely used to describe dietary patterns in various populations, including older adults [16,17]. Evidence indicates that an integrated approach, assessing health status, dietary intake, and health behaviors, can enhance our understanding of complex health outcomes [16,18]. Principal component analysis (PCA), factor analysis (FA), and cluster analysis (CA) are commonly applied to establish empirical dietary patterns [16,19]. These methods differ conceptually and methodologically in how they construct variables to determine dietary patterns or identify associations with diseases [19]. PCA is often appropriate for dimension reduction and the identification of key factors, FA can reveal interpretable patterns through latent factors, and CA is particularly suitable for identifying groups of elderly people with similar characteristics [17,20-22]. In Korea, few studies have sought to characterize older adults by applying multiple statistical methods to the same dataset and directly comparing the results [10,15]. In the present study, we analyzed data from a representative sample, whereas prior investigations have focused on specific subsets of older adults: one included participants from a local community [15], and another involved patients hospitalized at a regional university center [10].

The objective of this study was to compare the results derived from 3 analytical techniques employed to categorize profiles of older adults. These profiles were based on demographic characteristics, health status, physical condition, and health behaviors, with expansion through the inclusion of available dietary intake data. The results may provide a basis for prioritizing personalized nutrition plan formulation for individuals and groups.

## MATERIALS AND METHODS

### Data sources and study population

This study utilized data from the 8th Korea National Health and Nutrition Examination Survey (KNHANES VIII-1). This was the most recent iteration of the KNHANES and spanned the years 2019 to 2021. However, to evaluate frailty within the physical function domain, our analysis incorporated variables such as grip strength, which were present in the data from 2019 alone [23]. Therefore, we selected the 2019 data for this study. The respondents comprised 1,735 elderly individuals aged 65 years or older who took part in a health interview. From this group, we excluded individuals lacking demographic information (n= 221) and nutritional information (n= 153), as well as those with extreme energy intake values (under 400 or 5,000 kcal or greater, n= 9). After these exclusions, a total of 1,352 participants remained and were included in the analysis.

### Measurement and definition

In this study, we aimed to better understand the characteristics of older adults in Korea by establishing key concepts or components. Our framework was based on the position paper of the Academy of Nutrition and Dietetics [24] and the textbook *Epidemiology of Aging* [25]. Accordingly, the study incorporated demographic factors, health status, physical and functional status, cognition, and environmental factors as components potentially influencing nutritional status among older adults.

The demographic factors analyzed were age (treated as a continuous variable), sex (categorized as male or female), and education level (classified as “primary school or less,” “middle school,” or “high school, college, or higher”). Health status was assessed by considering the number of chronic diseases diagnosed; body mass index (BMI), calculated by dividing weight (in kilograms) by the square of height (in meters); subjective health status; and oral health (including subjective oral health status, self-perceived chewing discomfort, and oral pain).

Physical/functional status was assessed based on 3 criteria: physical discomfort, physical dysfunction, and subjective dizziness (all yes-or-no items). The cognitive component encompassed depression (yes or no), perceived stress (yes or no), and health/nutrition-related beliefs, which included perceived body shape (rated on a 5-point Likert scale) and weight control (yes or no).

Environmental factors considered in this study included residential area (urban, suburban, or rural), economic status (household income and economic activity), social connectedness, and lifestyle attributes such as smoking status, alcohol consumption, sleep duration, physical activity (PA) level, use of nutrition labels (yes or no), dietary habits, and habitual dietary intake data. Social connectedness was examined through measures of social support and social eating. Social support was assessed using marital status (either married or separated/divorced/widowed), family structure (1, 2, or 3 generations in the household), and the number of family members. Social eating was characterized by the practice of eating with others and the frequency of shared meals throughout the week [26].

In terms of lifestyle, smoking status and alcohol consumption were classified into 3 categories based on self-reported answers: never, former, or current. Sleep duration was categorized into 3 groups: short (≤ 6 hr/day), appropriate (7-9 hr/day), and long (≥ 10 hr/day) [27]. PA was assessed using self-reported responses to the KNHANES Global Physical Activity Questionnaire [28]. Based on the PA guidelines published by the World Health Organization, high-level PA was defined as achieving at least 1,500 metabolic equivalent of task (MET)-min/wk, or over 3,000 MET-min/wk if at least 3 day/wk involved high-intensity exercise. Moderate PA was defined as achieving 600-1,500 MET-min/wk or 1,500-3,000 MET-min/wk if at least 1 day to 2 days included high-intensity exercise. Low-level PA was defined as not meeting the criteria for high-level or moderate PA, with activity levels under 600 MET-min/wk [29,30]. Dietary habits were assessed using meal frequency per week, frequency of eating out per week, and intake of dietary supplements. Meal frequency was categorized as 5-7 times, 3-4 times, 1-2 times, or 0 times, and the average meal frequency per week was then calculated. The frequency of eating out was converted into monthly occurrences, and the average monthly frequency was determined. Habitual dietary intake was evaluated based on energy, protein, carbohydrate, fat, and vegetable/fruit consumption using 24-hour dietary recall survey data. Participants were classified into quintiles based on their intake levels.

The definition and measurement of variables influencing aging and nutritional status required the integration of all items with similar meanings. This involved combining or reconfiguring 2 or more items to generate new variables. Continuous variables were transformed into either multi-categorical or binary variables, while multi-scale categorical variables were converted into binary variables. Following these procedures, a total of 86 variables were included in the initial analysis (Supplementary Material 1).

### Statistical analysis

This procedure involved using appropriate variables transformed through optimal scaling to handle categorical data included in the dataset. Optimal scaling converts the original levels of categorical variables into category quantifications such that the variance in the new variables is maximized [31]. The maximum total variance method was employed for optimal scaling [32], and this was implemented using the SAS PRINQUAL procedure [20]. Continuous data were standardized using PCA, FA, and CA. Standardization, or Z-normalization, transforms the data to display a mean of 0 and a standard deviation of 1 [17]. Missing data were addressed in the same manner as non-missing variables during optimal transformation via the PROC PRINQUAL procedure. PROC PRINQUAL is an analytical technique that applies the alternating least squares method to discover linear and non-linear transformations of variables that optimize the properties of the correlation or covariance matrix of the transformed variables [20,33]. Furthermore, PROC PRINQUAL simultaneously computes the optimal transformations for the non-missing values and estimates the missing values to minimize the squared error [34]. The estimation of these values began at the initial stage of optimization as follows: after setting initial values for non-missing variables, the missing values were replaced with the means. These served as initial estimates for the original value of the variable and the specified transformation type, according to sets based on the same scale [35].

Descriptive statistics were utilized to summarize the socio-demographic characteristics, health status, and dietary intake of the study participants. The KNHANES data were analyzed using a complex sampling design that accounted for stratification, clustering, and weights in an effort to represent the entire Korean population. Weighted percentages were estimated with PROC SURVEYFREQ, while weighted means were derived using PROC SURVEYMEANS to accommodate the features of the sampling design. All statistical analyses were performed using SAS version 9.4.1 (SAS Institute Inc., Cary, NC, USA). A significance level of p-value < 0.05 was adopted for all statistical tests.

The techniques employed in this study for profiling factors included PCA, FA, and CA. Initially, 86 variables were identified to measure concepts or components characterizing the profiles of Korean older adults from the KNHANES dataset. For dimensionality reduction using PCA, we applied criteria such as eigenvalues greater than 1.0, scree plot analysis, PCA loadings of 0.3 or higher, and a cumulative variance of at least 70% to select key factors [21]. We subsequently evaluated the interpretability of the results. Following dimensionality reduction with PCA, FA and CA were performed using 19 items from the questionnaire. The suitability of the dataset for FA was assessed using the Kaiser-Meyer-Olkin (KMO) measure (> 0.5) and the Bartlett test of sphericity [21,36]. Factors were extracted based on an eigenvalue greater than 1.0, factor loadings of 0.6 or higher, and a cumulative variance of at least 70% [20,36]. To ascertain the number of clusters for *k*-means clustering using the Ward linkage method, we considered the PCA and FA results, setting the range for k between 3 clusters to 5 clusters. The *k*-means clustering was repeatedly tested with various initialization methods, including random starts, maximum-minimum criteria, and random partitions, to improve the accuracy of the k number and the stability of the clusters [17,37]. Furthermore, the *k*-means algorithm was configured to execute 10,000 iterations, and a seed from the pseudorandom number generator was used to ensure consistent randomness each time the algorithm ran [38]. The optimal number of clusters was determined by evaluating model fit indices such as pseudo F, cubic clustering criterion, and R^2^, as well as the interpretability of the resulting clusters [20,38] (Supplementary Material 2).

### Ethics statement

Additionally, the present study obtained approval from the Institutional Review Board of Dankook University (DKU 2021-03-049).

## RESULTS

### General characteristics of the study participants

Table 1 presents the characteristics of the study participants. The mean age was 72.3 years, and a slight majority were women (57.9%). Regarding marital status, 66.1% of participants had spouses, while the remaining 33.9% were widowed, divorced, or separated. Among the participants, 83.5% had been diagnosed with 1 or more chronic diseases. In terms of dietary intake, the average total energy intake was 1,544.7± 20.1 kcal/day, with carbohydrate intake at 258.2± 2.1 g/day, protein intake at 53.6± 1.0 g/day, fat intake at 28.1± 0.8 g/day, and fruit and vegetable intake at 470.2±13.2 g/day.

### Principal component extraction and characterization of composition

**Figure 1.**
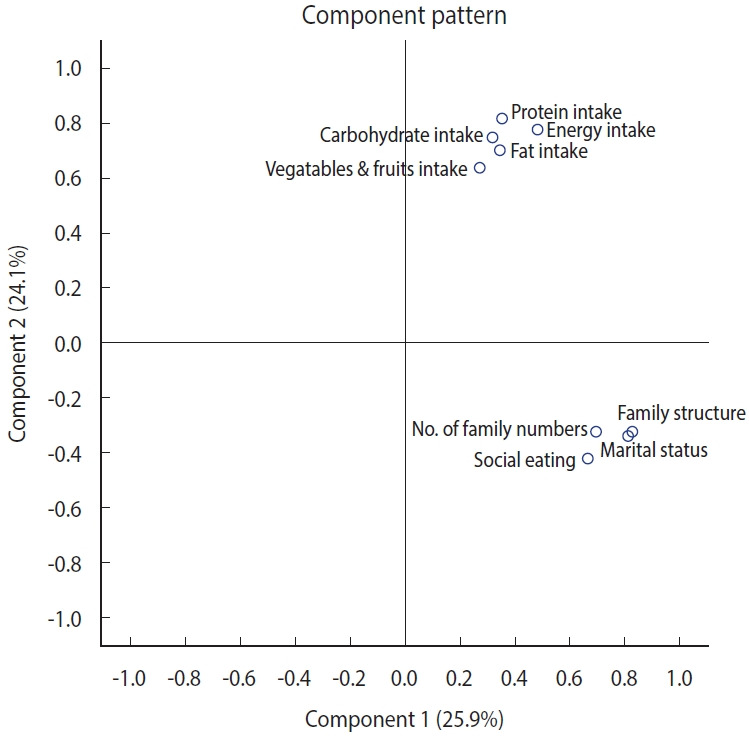
Principal components of a customized nutrition plan for Korean older adults.

Table 2 presents the results regarding the final dimensional model in PCA. Four principal components (PCs) were identified in the final model, which together explained approximately 72% of the total cumulative variance. The first PC represented social connectedness, including social support from family and social eating. The second PC was characterized by the dietary intake levels of the main nutrients. Oral health status and general health status were represented by the third and fourth PCs, respectively. Figure 1 illustrates the data distribution of Korean older adults as described by the first and second components through PCA, highlighting the key variables for each component.

### Factor analysis with extraction from major profiles

In FA, the KMO value was 0.51, indicating that the data were suitable for the analysis, and 5 factors were found to account for 74.3% of the total variance (Table 3). Factor 1 corresponded to a quantitative representation of major nutrient intake. Factor 2 captured characteristics of social connectedness among older adults, encompassing the number of family members, family structure, and engagement in social eating. Factor 3 pertained to oral health, including oral pain and subjective oral health status. Factor 4 related to physical health status, incorporating the number of chronic diseases and BMI. Factor 5 reflected perceived health status, which encompassed physical discomfort and subjective health status.

### *K*-means clustering of profiles

The CA results indicated that the model comprising 4 groups demonstrated the smallest distance of similarity among individuals within each group. Conversely, the distance of similarity between different groups was the largest (R^2^ = 0.465).

**Figure 2.**
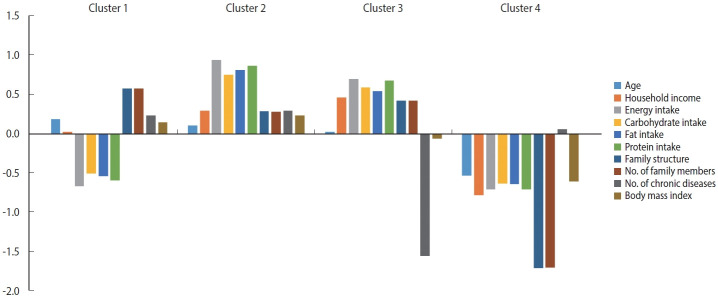
*K*-means clustering of profiles of Korean older adults. Dietary intake (energy, fat, carbohydrates, protein): quintile scale for each nutrient. The length of the bars in the graph indicates the relative cluster means of the 10 variables from a mean of 0 in each cluster. The cluster analysis results were analyzed using *k*-means clustering based on the transformed data. Data transformation incorporated the maximum total variance method for the optimal scaling of nominal and ordinal data, along with the z-score standardization method for continuous data.

The older adults in cluster 1 exhibited the greatest mean age and tended to received the most social support from their families. On average, this group displayed a relatively low household income and low nutrient intake, with most members diagnosed with multiple chronic diseases. The members of cluster 2 exhibited the greatest nutrient intake, while also having the highest rate of chronic diseases and the highest mean BMI. This cluster displayed a relatively high average age, strong family support, and a fairly high household income. In turn, cluster 3 exhibited the lowest number of chronic diseases and the highest household income. This group was characterized by a relatively young average age, high nutrient intake, strong social support from their families, and a comparatively low BMI. On average, cluster 4 included the youngest older adults, who displayed the lowest household income, nutrient intake, family support, and BMI (Figure 2).

Table 4 presents the key characteristics of the variables contributing to the formation of the 4 clusters. The mean ages for each cluster were as follows: cluster 1, 75.1± 4.7 years; cluster 2, 73.3± 5.0 years; cluster 3, 72.2± 5.1 years; and cluster 4, 71.0± 4.6 years. A significant difference in age was observed across clusters (F=28.0, p< 0.01). Approximately 50% to 60% of the individuals in clusters 1, 2, and 3 were categorized in the low and lower-middle income groups, while over 90% of cluster 4 fell into these income categories (*χ*^2^ = 179.0, p< 0.01). In clusters 1 and 4, around 60% of participants were in the first and second quintiles for total nutrient intake, while clusters 2 and 3 had proportions exceeding 50% in the fourth and fifth quintiles combined (energy: F= 918.3, p< 0.01; carbohydrates: F= 559.0, p< 0.01; fat: F= 585.8, p< 0.01; protein: F= 762.9, p< 0.01). Approximately 71% of individuals in cluster 4 lived alone, in contrast to those in clusters 1, 2, and 3, of whom about 90% lived with 2 or more family members (*χ*^2^ =971.3, p<0.01). Clusters 1, 2, and 4 had over 65% proportions of 2 or more chronic diseases, whereas cluster 3 consisted entirely of older adults without chronic diseases (*χ*^2^ = 945.0, p< 0.01).

## DISCUSSION

In this study, we classified characteristics of Korean older adults by incorporating domains influencing their quality of life and aging process into 3 analytical models: PCA, FA, and CA. The selection of these 3 multivariate analysis techniques was based on the intention to leverage the strengths of each [37] and to interpret the results through conceptually different methods [38]. PCA is a statistical technique for dimensionality reduction and exploratory analysis. This approach helps identify common trends and groupings among variables while minimizing information loss [39]. However, PCA has some drawbacks, such as subjectivity in selecting groups, determining the number of PCs or factors, and choosing factor loadings, as well as the pattern nomenclature [40,41]. FA is another statistical method that is used to extract underlying factors, also known as latent factors, from patterns of correlation among observed variables [21]. This technique uses the relationships between variables to uncover hidden factors inherent in the data. These factors, while not directly observable in the dataset, are essential for explaining variability [37]. CA is a method for classifying participants into distinct groups based on measured variables; each individual belongs to only 1 cluster, resulting in mutually exclusive patterns [17]. This classification enables the examination of relationships between subgroups and health outcomes or other characteristics, and it can also uncover subgroups at nutritional risk [16,37]. In contrast to CA, PCA, and FA do not produce mutually exclusive patterns. Instead, each participant is assigned a score that reflects adherence to each derived pattern. These scores are then used in subsequent analyses to explore associations between patterns and specific outcomes [31,42].

In the present study, the procedures for PCA, FA, and CA were conducted in accordance with the prescribed analysis steps and acceptance criteria [21,36-38]. The collected data were deemed appropriate for application based on the correlation matrix, the KMO value, and the Bartlett test of sphericity. Furthermore, the analysis results revealed high loadings for the profiling domains of older adults.

In this study, we attempted to integrate various factors from different domains to characterize the examined population. In nutritional epidemiology, a handful of studies have adopted a similar approach, focusing primarily on adolescents and young adults [13,39]. However, only a select few have employed multiple analytical techniques to thoroughly examine the characteristics of older adults [10,15]. Our research uncovered several significant findings in the profiling analysis of Korean older adults regarding customized nutrition planning. The analytical approaches identified 3 predominant factors: dietary intake, social support from family, and health status. These domains observed in the present study align with those identified previously. Kim & Hwang [15] classified Korean older adults into 4 groups—active seniors, active seniors at risk, non-active seniors, and non-active seniors at risk—based on dietary intake, family support, health status, and level of independent living. Ju & Chol [10] broadened the profiling domains to include lifestyle factors such as smoking and drinking, which are associated with the nutrition and health status of older adults.

In this investigation, the PCA and FA results comprised highly similar variables and factors. PCA extracted 4 components, while FA yielded 5 factors. Factors related to social connectedness, dietary intake, and oral health were consistent across both methods. In the PCA, subjective health, physical/functional status, and the number of chronic diseases were grouped into a single composite factor representing health status. In contrast, FA differentiated health status into 2 categories: objective indicators (such as the number of chronic diseases and BMI), which represented physical health status, and subjective indicators (including physical discomfort and subjective health perception), which reflected perceived health status. Some authors have posited that when common variances for most variables exceed 0.60 and the error (unique variance) is nearly zero, FA and PCA can yield similar results [19]. Our findings support this, as PCA and FA produced similar estimates due to the high common variance among the variables, with values of 0.71 and 0.69, respectively. The CA results also highlighted meaningful factors derived from PCA and FA. However, demographic variables such as age, sex, and household economic status were initially excluded from PCA and FA because of their low factor loading values. Subsequently, age and household economic status emerged as significant factors for classifying Korean older adults in the CA results. Some studies comparing PCA, FA, and CA procedures have reported analogous results [16-18]. Furthermore, previous research on aging profiles has confirmed that socioeconomic domains—including sex, age, economic status, and education level—are pivotal in explaining the characteristics of older adults [40-43].

In the domain of social connectedness, both PCA and FA results indicated that social support from the family and social eating were significant factors. However, family social support, which included the number of family members and the family structure, was identified as the only significant factor in the CA results. Factors such as marital status, form of cohabitation, household size, and the presence of children and grandchildren were also relevant in differentiating aging profiles [13]. Kim & Hwang [15] recognized family social support as a key factor in classifying Korean seniors for dietary support services. Their research showed that seniors living with a spouse displayed the best nutritional status, while those living alone exhibited the poorest. Similarly, Park et al. [13] observed that among the elderly, the use of convenience meals varied depending on whether children lived with them, regardless of meal type preference. This finding suggests that the presence of children influenced the family’s meal choices. Findings confirm that despite the diminished role of the traditional family in Korean society, social support within the family remains important for older adults [42]. Consequently, older adults living alone, without a spouse or children, should be provided with greater access to balanced meals through customized nutrition plans [43]. It is also vital to offer them ongoing nutrition education to promote a healthy diet within the community [12].

In this report, within the CA findings, Korean older adults were categorized into 4 distinct typologies. Cluster 3 exhibited a relatively young mean age, with positive profiles across all domains. Cluster 4 represented the youngest age group overall, yet they exhibited negative profiles across all domains. Cluster 1 comprised the oldest participants on average, with poor nutrition intake, poor health status, and low income, while also receiving the most social support. Cluster 2 exhibited the highest nutrition intake but comparatively poor health status, along with moderate income and social support. The findings of this study clearly indicate that Korean older adults are not a homogeneous group [8]; rather, substantial differences exist in terms of nutrition and health status. These results suggest that incorporating multi-domain characteristics into nutrient intake profiling could improve the identification of intermediate and high-risk groups for aging-related outcomes and facilitate the targeting of early interventions. Previous studies have similarly utilized profiling or clustering analysis to identify health-promoting behaviors, cognitions, and social activities as key elements of policy development for older adults [8,14]. Furthermore, these profiling results can serve as preliminary data for follow-up studies [5,14,44]. The profiles could also be employed in subsequent analyses to explore factors associated with age-related diseases and quality of life among older adults.

This study had several limitations. First, the results derived from the analytical technique employed for the profiling procedure are not generalizable. The multivariate analysis in this study utilized PCA, FA, and CA, each comprising several sub-analysis models. Notably, *k*-means clustering, the most widely used approach in CA, was employed. However, other methods such as the k-medoids algorithm and various hierarchical clustering models also exist. Therefore, we recommend that future research employ a broader and more comparable array of statistical models to conduct the profiling of older adults. Second, the participants were not categorized into age-based subgroups, and the analysis was conducted exclusively among the older population aged 65 years and above. Consequently, it was not feasible to compare the findings across different age groups.

In conclusion, this study delineated profiling domains to characterize and classify older adults in Korea, employing PCA, FA, and CA. The profiling domains identified through this exploratory study could inform intervention programs targeting nutritional issues, as well as future studies investigating the mechanisms underlying aging-related diseases and the deterioration of physical function that impact quality of life in older adults.

## Figures and Tables

**Figure f3-epih-46-e2024043:**
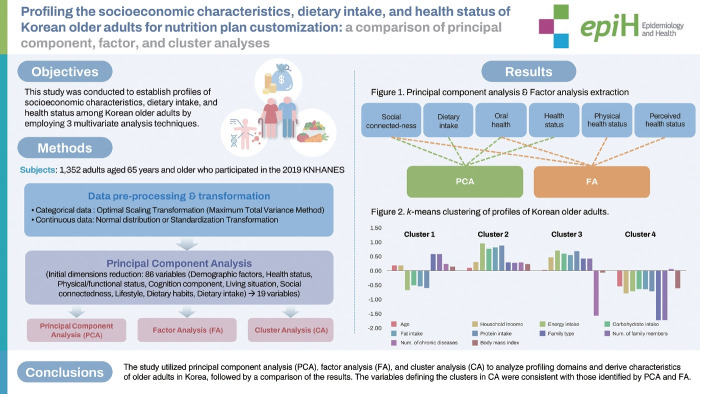


**Table 1.** General characteristics of study participants

Characteristics	Categories	Weighted %±SE or mean±SE
Age (yr)		72.3±0.2
Sex	Male	42.1±1.5
	Female	57.9±1.5
Education	Primary school or less	31.1±1.9
	Middle school	19.2±1.3
	High school, college, or higher	49.1±1.9
Marital status	Married	66.1±1.8
	Separated, divorced, or widowed	33.9±2.7
No. of family members	1	20.0±1.3
	2	52.2±1.8
	≥3	27.8±3.4
Household income	Low	11.3±1.5
	Lower-middle	20.4±1.3
	Middle	19.2±1.3
	Upper-middle	38.9±1.6
	High	10.3±1.1
Health status	No. of chronic diseases	
	0	6.5±1.2
	1	23.5±1.4
	≥2	60.0±2.7
	BMI, mean±SD (kg/m^2^)	24.1±0.1
Dietary intake	Total energy (kcal/day)	1,544.7±20.1
	Carbohydrate (g/day)	258.2±2.1
	Protein (g/day)	53.6±1.0
	Fat (g/day)	28.1±0.8
	Vegetables and fruits (g/day)	470.2±13.2

SE, standard error; BMI, body mass index; SD, standard deviation.

**Table 2.** Principal component extraction and characterization of composition

Components	Variables^1^	PCA loadings^2^	Eigenvalue	AV (%)
CP1 (social connectedness)	Marital status	0.399	3.883	25.9
	Social eating	0.358		
	Family structure	0.417		
	No. of family members	0.406		
CP1 (dietary intake)	Energy intake	0.419	3.622	50.0
	Protein intake	0.374		
	Carbohydrate intake	0.340		
	Fat intake	0.339		
	Vegetable and fruit intake	0.309		
CP3 (oral health)	Oral pain	0.632	1.782	61.9
	Subjective oral health status	0.549		
CP4 (health status)	Subjective health status	0.576	1.454	71.6
	Physical/functional status	0.513		
	No. of chronic diseases	0.368		

PCA, principal component analysis; CP1, first (main) component; CP2, second component; CP3, third component; CP4, fourth component; AV, accumulated variance.

1 Fifteen variables were identified as 4 principal components in the final model based on fit criteria of eigenvalues (>1.0), PCA loadings (≥0.3), and cumulative variance (≥70%).

2 PCA loadings (absolute value ≥0.30) for simplified results.

**Table 3.** Factor analysis with extraction from major profiles of Korean older adults

Factors	Variables^1^	Factor loadings^2^	Eigenvalue	Factor name
Factor 1	Energy intake	0.910	3.516	Dietary intake
	Protein intake	0.879		
	Carbohydrate intake	0.824		
	Fat intake	0.769		
	Vegetable and fruit intake	0.703		
Factor 2	Family structure	0.959	2.627	Social connectedness
	No. of family members	0.961		
	Social eating	0.796		
Factor 3	Oral pain	0.971	1.916	Oral health
	Subjective oral health status	0.918		
Factor 4	No. of chronic diseases	0.802	1.405	Physical health status
	Body mass index	0.723		
Factor 5	Physical discomfort	0.911	1.380	Perceived health status
	Subjective health status	0.690		

1 Factor loadings ≥|0.60| were considered significant.

2 Fourteen variables were identified as 5 factors in the final model based on fit criteria, including eigenvalues (>1.0), factor analysis loadings (≥0.6), and cumulative variance (≥70%).

**Table 4.** Characteristics of Korean older adults in cluster analysis^1^

Characteristics	Cluster 1 (n=502)	Cluster 2 (n=448)	Cluster 3 (n=154)	Cluster 4 (n=248)	χ^2^/F
Age (yr)	75.1±4.7	73.3±5.0	72.2±5.1	71.0±4.6	28.0^**^
Household income					179.0^**^
Low	200 (39.8)	120 (26.8)	31 (20.1)	176 (71.0)	
Lower-middle	145 (28.9)	153 (34.2)	82 (33.1)	54 (21.8)	
Middle	74 (14.7)	84 (18.8)	107 (16.2)	10 (4.0)	
Upper-middle	55 (11.0)	54 (12.1)	135 (18.2)	6 (2.4)	
High	28 (5.6)	37 (8.3)	154 (12.3)	2 (0.8)	
Dietary intake					
Energy intake					918.3^**^
1Q	187 (37.3)	0 (0.0)	1 (0.7)	82 (33.1)	
2Q	191 (38.1)	1 (0.2)	11 (7.1)	69 (27.8)	
3Q	111 (22.1)	60 (13.4)	38 (24.7)	60 (24.2)	
4Q	12 (2.4)	178 (39.7)	48 (31.2)	33 (13.3)	
5Q	1 (0.2)	209 (46.7)	56 (36.4)	4 (1.6)	
Carbohydrate intake					559.0^**^
1Q	167 (33.3)	13 (2.9)	5 (3.3)	86 (34.7)	
2Q	165 (32.9)	31 (6.9)	20 (13.0)	54 (21.8)	
3Q	111 (22.1)	72 (16.1)	32 (20.8)	60 (24.2)	
4Q	52 (10.4)	137 (30.6)	44 (28.6)	32 (12.9)	
5Q	7 (1.4)	195 (43.5)	53 (34.4)	16 (6.5)	
Fat intake					585.8^**^
1Q	184 (36.7)	3 (0.7)	5 (3.3)	80 (32.3)	
2Q	149 (29.7)	30 (6.7)	23 (14.9)	65 (26.2)	
3Q	109 (21.7)	72 (16.1)	33 (21.4)	57 (23.0)	
4Q	50 (10.0)	150 (33.5)	40 (26.0)	32 (12.9)	
5Q	10 (2.0)	193 (43.1)	53 (34.4)	14 (5.7)	
Protein intake					762.9^**^
1Q	181 (36.1)	1 (0.2)	1 (0.7)	88 (35.5)	
2Q	180 (35.9)	11 (2.5)	17 (11.0)	61 (24.6)	
3Q	101 (20.1)	73 (16.3)	35 (22.7)	61 (24.6)	
4Q	38 (7.6)	164 (36.6)	39 (25.3)	29 (11.7)	
5Q	2 (0.4)	199 (44.4)	62 (40.3)	9 (3.6)	
Family structure					981.7^**^
One generation alone	56 (11.2)	27 (6.0)	18 (11.7)	1 (0.4)	
Two generations	137 (27.3)	362 (81.0)	126 (81.8)	6 (2.4)	
Three generations	309 (61.5)	59 (13.0)	10 (6.5)	241 (97.2)	
No. of family members					971.3^**^
1	33 (6.5)	59 (13.2)	3 (2.0)	177 (71.3)	
2	143 (28.6)	282 (63.0)	150 (97.2)	71 (28.7)	
≥3	326 (64.9)	107 (23.9)	1 (0.8)	0 (0.0)	
No. of chronic diseases					845.0^**^
0	24 (7.2)	0 (0.0)	154 (100)	36 (9.7)	
1	49 (26.1)	138 (30.8)	0 (0.0)	131 (19.8)	
≥2	175 (66.7)	310 (69.2)	0 (0.0)	335 (70.6)	
BMI (kg/m^2^)	24.1±3.3	24.6±3.0	23.8±3.4	22.9±2.6	10.5^**^

Values are presented as mean±standard deviation of number (%). Q, quantile scale; BMI, body mass index.

1 Results of analyzing the characteristics of clusters of Korean older adults are based on original data; Ten variables were classified into 4 clusters based on fit criteria (R2, pseudo F, cubic clustering criterion, R2 for variables) in the cluster analysis.

** p<0.01 by chi-square test and one-way analysis of variance.

## Supplementary materials

Supplementary Material 1. The 86 variables included in the initial analysis.

Supplementary Material 2. Flow chart of data preprocessing and statistical analysis

## References

[1] Amarya S, Singh K, Sabharwal M (2015). Changes during aging and their association with malnutrition. J Clin Gerontol Geriatr.

[2] Lee S (2023). Outlook and challenges of population policy in 2023. Health Welf Forum.

[3] Norman K, Haß U, Pirlich M (2021). Malnutrition in older adults-recent advances and remaining challenges. Nutrients.

[4] Cristina NM, Lucia D (2021). Nutrition and healthy aging: prevention and treatment of gastrointestinal diseases. Nutrients.

[5] Vajdi M, Farhangi MA (2020). A systematic review of the association between dietary patterns and health-related quality of life. Health Qual Life Outcomes.

[6] Oh C, No JK, Kim HS (2014). Dietary pattern classifications with nutrient intake and body composition changes in Korean elderly. Nutr Res Pract.

[7] Gang G, Lee MJ, Choi EH, Lee HL, Lee HY, Chang HJ (2023). Evaluation on the nutrition quotient scores of elderly people living alone in Korea. Nutrients.

[8] Lee Y (2021). Activity profiles among older adults: latent class analysis using the Korean Time Use Survey. Int J Environ Res Public Health.

[9] Kim KB, Eun SJ (2019). Classification of clusters, characteristics and related factors according to drinking, smoking, exercising and nutrition among Korean adults. J Korea Acad Ind Coop Soc.

[10] Ju E, Choi J (2017). Identifying latent classes of risk factors for coronary artery disease. J Korean Acad Nurs.

[11] Keum YB, Yu QM, Seo JS (2012). Nutritional status and metabolic syndrome risk according to the dietary pattern of adult single-person household, based on the Korea National Health and Nutrition Examination Survey. J Nutr Health.

[12] Oh JH, Jung BM (2019). Comparison analysis of dietary behavior and nutrient intakes of the elderly according to their family status: the Korea National Health and Nutrition Examination Survey 2013-2016. Korean J Community Nutr.

[13] Park JY, Kim JN, Hong WS, Shin WS (2012). Survey on present use and future demand for the convenience food in the elderly group. Korean J Community Nutr.

[14] Park KH, Park JH (2019). Analysis of convergent influence of functional level, environmental factors and lifestyle on health and quality of life among elderly using structural equation model. J Korea Converg Soc.

[15] Kim JS, Hwang EM (2017). Support plan for providing customized meal services according to the characteristics of the elderly. Health Welf Issue Focus.

[16] Hearty AP, Gibney MJ (2009). Comparison of cluster and principal component analysis techniques to derive dietary patterns in Irish adults. Br J Nutr.

[17] Sauvageot N, Schritz A, Leite S, Alkerwi A, Stranges S, Zannad F (2017). Stability-based validation of dietary patterns obtained by cluster analysis. Nutr J.

[18] Cunha DB, Almeida RM, Pereira RA (2010). A comparison of three statistical methods applied in the identification of eating patterns. Cad Saude Publica.

[19] Bakolis I (2013). The use of dietary patterns empirically derived from principal components analysis and alternative strategies to identify associations between diet and disease [dissertation].

[20] https://support.sas.com/resources/papers/proceedings18/1844-2018.pdf.

[21] Santos RO, Gorgulho BM, Castro MA, Fisberg RM, Marchioni DM, Baltar VT (2019). Principal component analysis and factor analysis: differences and similarities in nutritional epidemiology application. Rev Bras Epidemiol.

[22] Kim KY, Yun JM (2021). Dietary patterns and mild cognitive impairment risk in Korean adults over 50 years old. Prev Nutr Food Sci.

[23] https://knhanes.kdca.go.kr/knhanes/sub04/sub04_04_01.do.

[24] Bernstein M, Munoz N, Academy of Nutrition and Dietetics (2012). Position of the Academy of Nutrition and Dietetics: food and nutrition for older adults: promoting health and wellness. J Acad Nutr Diet.

[25] Korean Society of Epidemiology (2012). Textbook of epidemiology of aging.

[26] Dunbar RI (2017). Breaking bread: the functions of social eating. Adapt Human Behav Physiol.

[27] Fang J, Wheaton AG, Keenan NL, Greenlund KJ, Perry GS, Croft JB (2012). Association of sleep duration and hypertension among US adults varies by age and sex. Am J Hypertens.

[28] Sung H, Kim G, Ma X, Choe H, Han Y, Yoon J (2022). Physical activity trends in Korean adults from Korea National Health and Nutritional Examination Survey from 2014 to 2019. Int J Environ Res Public Health.

[29] https://www.who.int/publications/m/item/global-physicalactivity-questionnaire.

[30] Macek P, Terek-Derszniak M, Zak M, Biskup M, Ciepiela P, Krol H (2019). WHO recommendations on physical activity versus compliance rate within a specific urban population as assessed through IPAQ survey: a cross-sectional cohort study. BMJ Open.

[31] Young FW (1981). Quantitative analysis of qualitative data. Psychometrika.

[32] Meulman JJ, Van der Kooij AJ, Heiser WJ (2004). In: Kaplan D. The SAGE handbook of quantitative methodology for the social sciences.

[33] Young FW, Takane Y, de Leeuw J (1978). The principal components of mixed measurement level multivariate data: an alternating least squares method with optimal scaling features. Psychometrika.

[34] Mayer B, Muche R, Hohl K (2012). Software for the handling and imputation of missing data-an overview. J Clinic Trials.

[35] SAS Institute (1993). SAS technical report R-108: algorithms for the PRINQUAL and TRANSREG procedures.

[36] Shrestha N (2021). Factor analysis as a tool for survey analysis. Am J Appl Math Stat.

[37] Maugeri A, Barchitta M, Favara G, La Mastra C, La Rosa MC, Magnano San Lio R (2022). The application of clustering on principal components for nutritional epidemiology: a workflow to derive dietary patterns. Nutrients.

[38] Fränti P, Sieranoja S (2019). How much can k-means be improved by using better initialization and repeats?. Pattern Recognit.

[39] Choi HJ, Joung H, Lee HJ, Jang HB, Kang JH, Song J (2011). The influence of dietary patterns on the nutritional profile in a Korean child cohort study. Osong Public Health Res Perspect.

[40] Thorpe MG, Milte CM, Crawford D, McNaughton SA (2016). A comparison of the dietary patterns derived by principal component analysis and cluster analysis in older Australians. Int J Behav Nutr Phys Act.

[41] Linting M, Meulman JJ, Groenen PJ, van der Koojj AJ (2007). Nonlinear principal components analysis: introduction and application. Psychol Methods.

[42] Han SW (2013). Socio-demographic inequalities of social support in South Korea. Korean J Soc Issues.

[43] Lee YJ, Kwon MK, Baek HJ, Lee SS (2015). Comparative analysis of food intake according to the family type of elderly women in Seoul area. J Nutr Health.

[44] Kim J, Lee Y, Won CW, Kim MK, Kye S, Shim JS (2021). Dietary patterns and frailty in older Korean adults: results from the Korean frailty and aging cohort study. Nutrients.

