# Supplementary material for: Profiling the socioeconomic characteristics, dietary intake, and health status of Korean older adults for nutrition plan customization: a comparison of principal component, factor, and cluster analyses
Source: Epidemiol Health. 2024 Apr 12;46:e2024043. doi: 10.4178/epih.e2024043 (PMC11417451; doi:10.4178/epih.e2024043)
Supplement: Supplementary Material 1. — The 86 variables included in the initial analysis. [file epih-46-e2024043-Supplementary-1.docx]

Supplementary Material 1. The 86 variables included in the initial analysis.

| **Demographic factors** | **Health status** | **Physical/functional status** | **Cognition component** | **Dietary habits** |
| --- | --- | --- | --- | --- |
| Age (yr)  Age (5scale)  sex  education | family history of chronic diseases  chronic diseases  number of chronic diseases  body mass index  weight  height  subjective health status  subjective oral health status  self-perceived chewing discomfort  oral pain  abdominal obesity  obesity | physical discomfort  physical dysfunction  subjective dizziness  hearing problems  olfactory dysfunction  vision problems  sensory organ diseases  number of sensory organ diseases  body balance  grip Strength  quality of life | depression (Y/N)  diagnosis of depression  treatment for depression  perceived stress  body shape (5scale)  weight control  subjective body image  efforts for weight control | meal frequency  eating out frequency  breakfast Frequency  lunch Frequency  dinner Frequency  dietary supplements intake  nutrition education & counseling  using food label  belief in nutrition labels  use of nutrition labeling  awareness about nutrition labels  Impact of nutrition labels |
| **Living situation** | **Social support** | **Lifestyle**  **(Smoking & Alcohol)** | **Lifestyle**  **(Physical Activity, PA)** | **Dietary intakes** |
| region  economics (y/n)  economic activity  household Income (5 levels)  household Income (3 levels)  occupational Status | marital status  family type  number of family members  marital Status  number of Family Members (law)  number of Household members (1, 2, above 3)  household Composition | lifetime smoking  current smoking  daily average smoking -amount  nicotine dependency  smoking Period for former smokers  frequency of Binge drinking  alcohol Consumption frequency  adequate Sleep  average Sleep Duration | MET-min/week  total PA  PA (Y/N)  PA work time  PA transportation time  PA leisure time  type of PA  walking time  frequency of muscle  strengthening activities  weight Change  number of days with strength training  sitting time  decrease in walking activity  decrease in Physical activity | energy total intakes  protein total intakes  carbohydrate total intakes  fat total intakes  vegetables & fruits total intakes  energy EER  energy 5quintiles  energy 4quintiles  carbohydrate EER  carbohydrate 5quintiles  carbohydrate 4quintiles  fat EER  fat 5quintiles  fat 4quintiles  protein EER  protein 5quintiles  protein 4quintiles  vegetables & Fruits 4quintiles  Vegetables & Fruits 5quintiles |
|  | **social eating** |  |  |  |
|  | having meal with others  having Breakfast with others  having lunch with others  having dinner with others |  |  |  |
