# Supplementary material for: Profiling the socioeconomic characteristics, dietary intake, and health status of Korean older adults for nutrition plan customization: a comparison of principal component, factor, and cluster analyses
Source: Epidemiol Health. 2024 Apr 12;46:e2024043. doi: 10.4178/epih.e2024043 (PMC11417451; doi:10.4178/epih.e2024043)
Supplement: Supplementary Material 2. — Flow chart of data preprocessing and statistical analysis [file epih-46-e2024043-Supplementary-2.docx]

Supplementary Material 2. Flow chart of data preprocessing and statistical analysis
